# Cognitive skills of emergency medical services crew members: a literature review

**DOI:** 10.1186/s12873-020-00330-1

**Published:** 2020-05-29

**Authors:** Martin Sedlár

**Affiliations:** Institute of Experimental Psychology, Centre of Social and Psychological Sciences, Slovak Academy of Sciences, Dúbravská cesta 9, 841 04 Bratislava, Slovak Republic

**Keywords:** Emergency medical services, Ambulance, Prehospital, Non-technical skills, Cognitive skills, Situation awareness, Decision making, Literature review

## Abstract

**Background:**

Situation awareness and decision making, listed in non-technical skills taxonomies, are critical for effective and safe performance in high-risk professions. These cognitive skills and their behavioral markers have been studied less in emergency medical services (EMS) crew members. This paper aims to review the existing literature and identify important aspects and behavioral markers of situation awareness and decision making in EMS crew members – those who work in the role of prehospital emergency care providers – and to synthesize findings as a basis for developing a rating and training tool.

**Method:**

The search for relevant articles was conducted using electronic databases, reference lists of relevant reviews and included articles and personal collection of articles. The selection process based on the PRISMA statement yielded a total of 30 articles that met the eligibility criteria. Their findings were qualitatively synthesized using the structured approach, informed by the already known structure: situation awareness and its elements (gathering information, interpreting information, anticipating future states), decision making and its elements (generating and considering options, selecting and implementing an option, reviewing outcome/decision). Moreover, the element of maintaining standards also emerged as highly relevant for cognitive skills.

**Results:**

This review found an increased research interest in the non-technical cognitive skills of EMS crew members. The majority of included articles’ research designs were qualitative, then mixed, Delphi, and quantitative. It revealed several specifics of cognitive skills, such as EMS crew members need to holistically assess a wide range of cues and information, to make various health- and safety-related decisions and take EMS standards into account. However, there was only a limited number of observable markers of cognitive skills, such as acts and verbalizations, that could be considered as examples of good behavior. In addition, findings indicate a lack of articles focused on mass-casualty incidents and the interconnection of cognitive skills with other non-technical and medical skills.

**Conclusion:**

Further research is needed to get a more comprehensive view of behavioral markers of cognitive skills and to develop a rating and training tool to improve EMS crew members’ cognitive performance.

## Background

Situation awareness and decision making are paramount for emergency medical services (EMS) crew members in encountering and managing various routine and non-routine situations [1] with high stakes, complexity, dynamic changes, multiple stimuli, uncertainty, stress, and the high likelihood of being error-prone [2]. These cognitive skills are on the list of key non-technical skills. If they are satisfactory, they enable safe and quality prehospital emergency care [3–5]. Since human error is in the spotlight as the main contributing factor in poor safety outcomes [6], the significance of studying cognitive and other non-technical skills has become more apparent. It is demonstrated in skills taxonomies, behavior rating tools, and skills trainings [7–11].

Situation awareness, defined as knowing what is happening in the environment, is based on three elements: gathering information, interpreting information, and anticipating future states [3, 12]. Sometimes the term ‘situation awareness’ interchanges with the term ‘situation assessment’ [13]. A comparison of theories suggests that both terms relate to the understanding of the situation and have analogical cognitive processes [12–14]. For that reason, these terms are here used together under the category of situation awareness. Considering the interconnection between situation awareness and decision making [1, 15], situation awareness is the foundation for decision making. The non-technical skills category of decision making – simply viewed as reaching a judgment or choosing an option – contains four elements: situation assessment/defining problem, generating and considering options, selecting and implementing an option, and outcome review [3]. As seen, decision making goes beyond the act of the decision itself and situation assessment represents the first step in making decisions [13].

The defined cognitive skills can be incorrectly confused with medical and technical skills. Although both skills are cognitive in their essence – requiring a form of cognitive activity – indeed they differ. Medical and technical skills refer to specific elements of medical diagnosis, treatment, and the physical procedures thereof [16]. In other words, they are about using medical expertise, drugs, and equipment [11]. In contrast, non-technical cognitive skills are conceptualized as general skills [17], referring to diverse situational elements that must be perceived, monitored, assessed, and decided upon, without the need for manual dexterity. Despite the difference, non-technical skills complement technical skills [3]. Besides, there is evidence of their correlation [18, 19].

Cognitive skills and their individual processes have been scrutinized predominantly in naturalistic decision making and expertise research [20]. In general, they are harder to be directly observable in behavior, compared with social skills, because they occur primarily in the brain. However, the non-technical skills movement, which emphasizes the behavioral level of skills, claims that situation awareness and decision making can be inferred from some observable behavior, such as specific actions and verbalizations [21].

So far, the existing literature summaries has not brought enough details about cognitive skills in EMS crew members, and not at all about their behavioral markers. This shows a paucity of research on non-technical cognitive skills, expertise, and safety, which would assist in improving performance in the EMS field. Such research is also needed as each medical specialty has its specifics related to the personnel and work environment, and it is not correct to simply apply findings from one specialty to another.

This paper looks closer on cognitive skills, it aims to review the existing literature and identify important aspects and markers of situation awareness and decision making in EMS crew members – those who work in the role of prehospital emergency care providers – and to synthesize findings as a basis for developing a rating and training tool.

## Method

### Search method

This literature review utilized the Preferred Reporting Items for Systematic Reviews and Meta-Analysis (PRISMA) statement [22] and the Cochrane Handbook for Systematic Reviews of Interventions [23] to ensure methodological accuracy. Familiarization with the relevant literature on non-technical skills, naturalistic decision making, and expertise informed a process of creating the best search strategy, which would produce a limited number of irrelevant records and a multitude of relevant records. This process required the iterative cycle of searches using various synonyms and very close terms that were in line with the review aim. The final search strategy using Boolean operators contained the terms related to two main concepts: non-technical cognitive skills of situation awareness and decision making, and EMS crew members. The search for relevant articles was conducted in December 2018, without any publication date limitations, using electronic databases and additionally by hand. More details about the search strategy and information sources are given in Table 1.

**Table 1:** Information sources, search strategy, and inclusion/exclusion criteria

	Information sources	
Searching electronic databases	Scopus, Web of Science (Core Collection), Science Direct, EBSCO (MEDLINE, PsycINFO, CINAHL, Psychology and Behavioral Sciences Complete), and *ProQuest Central (Health and Medical Collection, Nursing and Allied Health Database, Psychology Database, Research Library, Science Database)*	
Hand-searching	Reference lists of relevant reviews and included articles, and personal collection of articles	
	Search strategy	
Non-technical cognitive skills	("non-technical" OR "nontechnical” OR "human factor*" OR "crew resource management" OR "crisis resource management" OR "competenc*" OR "skill" OR "skills" OR "situation awareness" OR "situational awareness" OR "situation assessment" OR "situational assessment" OR "sensemaking" OR "sense-making" OR "decision making" OR "decision-making") AND	
EMS crew members	("emergency medical service*" OR "paramedic*" OR "ambulance*" OR "prehospital" OR "pre-hospital" OR "out of hospital" OR "out-of-hospital" OR "emergency technician*" OR "emergency practitioner*" OR "emergency medical technician*" OR "emergency care practitioner*")	
	Inclusion criteria	Exclusion criteria
Language	Articles written in English	Articles not written in English
Publication type	Peer-reviewed research articles, review articles, and conference articles	Book chapters, editorials, abstracts, commentaries, letters, gray literature
Population	Articles containing data relevant to various EMS ambulance members, such as paramedics, physicians, nurses, emergency medical technicians, emergency care practitioners, providing prehospital emergency care	Articles containing data relevant only to EMS students, trainees, drivers, pilots, trainers, teachers, supervisors, managers, and professionals providing tactical, wilderness or hospital care
Topic	Articles containing data relevant to non-technical cognitive skills of situation awareness and decision making	Articles containing data relevant to technical skills and very specific medical cases.
Outcome	Articles containing data about important aspects and markers of good situation awareness and decision making	Articles containing data only about the importance of situation awareness and decision making, non-technical skills levels after some interventions, association between non-technical skills and other variables, and about all human, situational, and organizational factors influencing performance.

### Selection process

**Fig. 1:**
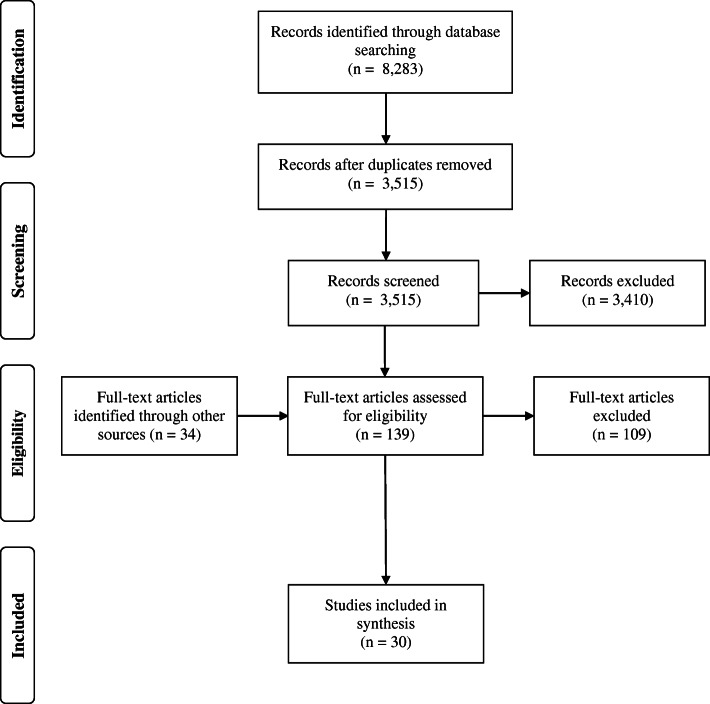
PRISMA flow diagram of the review process

Relevant articles had to meet the inclusion/exclusion criteria (see Table 1), encompassing key aspects and markers of non-technical cognitive skills applicable in EMS crew members. The first two criteria (articles written in English and peer-reviewed articles) were applied already in searching databases. The inclusion of only peer-reviewed articles was used as the quality indicator of articles. The search was limited to article titles and abstracts, but this was not possible in each database (Web of Science allows searching only ‘topics’, which includes titles, abstracts, keywords together – the same applies to Science Direct). After importing records into the reference manager Mendeley and removing duplicates by software and by hand, titles and abstracts were screened, and full texts of potentially relevant articles were retrieved. Additional full-text articles were identified through other sources, i.e., reference lists of relevant reviews [4, 24–28] and included articles, and personal collection of articles. Two reviewers independently reviewed 30% of records and full texts for eligibility; disagreements were resolved by discussion. This served to eliminate biases and take a consensual view on the selection process. Keeping this view in mind, one reviewer reviewed the remaining records and full texts. Ultimately, the selection process, displayed in Fig. 1 with the PRISMA flow diagram by Moher et al. [29], yielded 30 relevant full-text articles that met the selection criteria.

### Data extraction and synthesis

Data were extracted from full-text articles using a Microsoft Excel form with a pre-established list of data items with the following headings: first author and year, study location, design, sample, and relevant findings. The study location was derived from the first author’s country and the sample consisted of the number of participants included in the analysis – not all persons contacted to participate or excluded before analysis.

The extraction and synthesis of relevant findings were based on the theory of non-technical skills categories and elements [3] and the structured approach originally developed for the data analysis from the Critical Decision Method [30]. At first, reading articles served for better understanding and familiarization. Then, findings from each article were extracted and categorized according to their relevance to situation awareness and its elements (gathering information, interpreting information, anticipating future states), and to decision making and its elements (generating and considering options, selecting and implementing an option, reviewing outcome/decision). Reading articles indicated that the element of maintaining standards, originally attributed to the category of leadership [3], is an integral part of cognitive skills. For that reason, findings relevant to this element were also extracted. In the next stage, specific aspects and markers of each cognitive skill and element were identified in each article. In the end, all relevant findings from all articles were integrated. The synthesis of findings was organized linearly for clarity, however, in real emergency situations, the situation awareness phase overlaps with the decision-making phase. Furthermore, it must be noted that the synthesis depends entirely on qualitative data. In the case of articles with quantitative or other designs, only their quantitative, descriptive parts were used.

## Results

Of the 30 included research articles, the majority were qualitative in design (*n* = 17), then mixed (*n* = 5), Delphi (*n* = 5), and quantitative (*n* = 3). Their publication date ranged from 2003 to 2018. The study locations were Sweden (*n* = 8), United States (*n* = 5), Canada (*n* = 5), Slovakia (*n* = 3), United Kingdom (*n* = 2), Australia (*n* = 1), New Zealand (*n* = 1), Switzerland (*n* = 1), Norway (*n* = 1), Finland (*n* = 1), Iran (*n* = 1), and Taiwan (*n* = 1). The overview of the reviewed articles is given in Table 2.

**Table 2:** Overview of reviewed articles

First author and year	Study location	Aim	Design	Sample	Main findings relevant to situation awareness and decision making
Campeau 2009 [31]	Canada	To generate a substantive theory of paramedic scene management practice.	Qualitative: interviews	Paramedics (*n* = 24)	The space-control theory of paramedic scene management describes that paramedics coordinate key specific social processes (reducing uncertainty through social relations, controlling the trajectory of the scene, temporality at the scene, collateral monitoring) to establish “space control” at the scene. The space (or environment) is interpreted broadly to include both human and physical (non-human) elements.
Campeau 2011 [32]	Canada	To develop a theory about an important aspect of practice by exploring expertise in scene-management among paramedics.	Qualitative: interviews	Paramedics (*n* = 24)	The main category of paramedic expertise in scene management called paramedic kairotope means knowing when and where to act and acting at the right time and place. It is based on two subcategories: substantial use of subtleties in interpersonal communication, and innovative problem solving.
Chang 2018 [33]	Taiwan	To identify the perceived most desirable core competencies for all levels of emergency medical technicians.	Quantitative: statistical analysis of questionnaires	Emergency medical technician instructors (*n* = 84), emergency medical technician medical directors (*n* = 9)	Identified core competencies were related to assessing scene safety; conducting ongoing assessment; an accurate understanding of the patient from the medical and psychological point of view; proving appropriate care for each patient group and considering moral, social, and religious aspects of care; acting in accordance with ethics and health and safety policies.
Crowe 2017 [34]	United States	To describe the components of team leadership and team membership on a single patient call where multiple EMS providers are present.	Qualitative: focus groups	Paramedics (*n* = 9)	Key components of team leadership were: creating an appropriate action plan; receiving, processing, verifying and prioritizing information gained from team members; reconciling incongruent information; assessing situation and modifying plan. Key components of team membership were: being situationally aware, demonstrating appreciative inquiry; being safety conscious/advocating for safety.
Gurňáková 2013 [35]	Slovakia	To assess the frequency and the nature of deviations from recommended guidelines in paramedic teams in the selected competition task.	Mixed: videos, interviews, ratings of performance, statistical methods	Paramedics (*n* = 56)	Processing of initial information from the dispatch center of the most successful paramedic teams were characterized by paying full attention to initial information and forming an appropriate image of the situation. After arriving on the scene, they made more examinations recommended by guidelines and took more medical history data. They also verified verbal information by examinations and summarized gathered information after some intervals.
Gurňáková 2017 [36]	Slovakia	To test the relationship between performance confidence and actual performance in EMS crew leaders in the selected competition task.	Mixed: videos, interviews, ratings of performance, statistical methods	EMS physicians (*n* = 24), paramedics (*n* = 27)	The most successful EMS leaders’ performance confidence was as high as their actual performance. Their approach to gathering and interpreting information was characterized by collecting as much information as possible, making an initial hypothesis about the patient’s health problems, but verifying it by doing all necessary examinations, following medical guidelines properly.
Henderson 2013 [37]	United States	To examine difficult and complex service interactions between EMS providers and front-line patients.	Qualitative: interviews	Paramedics (*n* = 30)	Communication with the patient and information provided from bystanders’ behavior and communication, and assessment of the patient’s identity (a combination of race, gender, socioeconomic status, and potentially other variables) serve to shape street-level interaction between EMS and the patient.
Holly 2017 [38]	United Kingdom	To identify non-technical skills for use within a behavior rating system relevant to the provision of pre-hospital emergency care within rural/remote settings, and to develop and pilot the behavior rating system.	Mixed: literature review, observation of existing training course, expert advice, questionnaire	Development phase: experts in pre-hospital care or emergency medicine (*n* = 4), BASICS Scotland Course directors (n = 3), health psychologist (n = 2) Evaluation phase: general practitioners (*n* = 67), practice nurses (*n* = 9), paramedics (*n* = 2), participant who did not state their profession (*n* = 4)	The behavioral rating system includes one relevant category - gathering information. It is comprised of three elements and associated markers: conducting a risk assessment of the scene and ensuring it is safe to approach the patient before proceeding (e.g., noting any environmental hazards to self and the patient, stating the decision to approach the patient or not), reviewing decisions to ensure they are still appropriate requesting a second opinion from others (e.g., discussing alternative suggestions, stating the final decision), and trying alternative options when a certain approach is not working (e.g., stating alternative approach/intervention to be tried, beginning to use new approach/intervention).
Holmberg 2010 [39]	Sweden	To describe the registered nurses’ experience of being responsible for the care of the patient in the ambulance care setting.	Qualitative: interviews	Registered nurses (*n* = 5)	Important parts of the care for patients in ambulance services were encountering the patient unprejudiced and with no pre-made assumptions, but at the same time to have a plan; being open-minded and have a broader outlook at the situation; medical assessment and assessment of the patient’s and significant others’ psycho-social situation and needs; making medical and nursing decisions.
Holmberg 2017 [40]	Sweden	To identify the types of knowledge that EMS managers considered desirable in their ambulance clinicians.	Modified Delphi	Registered nurses and emergency medical technicians working as EMS managers: 1st round (*n* = 30) 2nd round (*n* = 27) 3rd round (*n* = 24)	The desired knowledge was related to following categories and sub-categories: knowledge to handle the specific ambulance care (e.g., knowledge to assess risks in a dangerous environment, knowledge to manage personal safety), knowledge to assess and care for patients’ medical needs (e.g., knowledge to assess and care for different medical conditions, knowledge of treatment guidelines), knowledge to holistically assess and care for patients (e.g., knowledge to assess the patient’s situation from a holistic perspective, knowledge to support persons in crisis).
Jensen 2011 [41]	Canada	To establish consensus on the most important clinical decisions paramedics make during high-acuity emergency calls and to visualize these decisions on a process map of an emergency call.	Delphi	Medical directors and paramedics, the latter of whom work a variety of roles (ground ambulance paramedic, supervisor or manager, quality assurance, clinical development, and educator): 1st round (*n* = 23) 2nd round (*n* = 22) 3rd round (*n* = 20) 4th round (*n* = 23)	Identified important decisions during high-acuity calls were related to scene management (e.g., recognize potential hazards and scene safety, decide when to leave the scene and when to treat o scene), assessment (e.g., initial assessments of the patient’ medical condition, decide if the patient has the capacity to refuse or content), and treatment (e.g., decide on appropriate treatment, reassess the patient after giving a treatment, decide to change care plan based on patient changes).
Kilner 2004 [42]	United Kingdom	To identify desirable attributes of the ambulance technician, paramedic, and clinical supervisor to inform future curriculum development.	Delphi	Chief executives and medical directors or advisors of ambulance trusts: 1st round (*n* = 34) 2nd round (*n* = 42)	Desirable attributes were identified: abilities to take a history and conduct patient assessment and examination of both adults and children; intellectual skills to enable the interpretation of clinical data, the implementation of clinical judgment and decision making and the formulation of a diagnosis; aware of and adherence to national and local guidelines and policies.
King 2010 [43]	United States	To identify attributes that distinguish effective from ineffective responders and leaders in a disaster.	Qualitative: focus groups	Medical directors for the 9-1-1 EMS systems (*n* = 24)	Attributes of effective disaster responders were grouped in following categories: disaster training and experience (e.g., can assess or “size up” the disaster situation), cognition (e.g., can weight or balance options, can focus and concentrate, can think quickly, think or plan ahead, sees the broader picture), problem solving/decision making (e.g., can make difficult decisions, considers all available information, can decide and act based in incomplete information), adaptable/flexible (e.g., able to change one’s way of doing things, considers alternatives, open-mindedness).
Myers 2016 [44]	New Zealand	To adapt and evaluate a non-technical skills rating framework for the air ambulance clinical environment.	Mixed: adaptation phase – expert working group, scoping review, focus groups, clinician survey, filed testing data; evaluation phase –assessments of video-recorded performance, assessments of performance immediately after simulations	Survey: clinicians (*n* = 38), flight nurses (*n* = 20), specialist transport physicians (*n* = 12), paramedics (*n* = 6) Simulation study: physicians from specialty training programs in intensive care and anesthesia (*n* = 3), anesthetics (*n* = 3), critical and intensive care medicine (*n* = 3), emergency medicine (*n* = 5), general medicine (n = 2)	Non-technical skills rating framework includes the following relevant categories, associated elements and examples of positive behaviors. Situation awareness referred to gathering information (e.g., conducting a frequent scan of the environment, cross-checking information to increase reliability), recognizing and understanding (e.g., increasing frequency of monitoring in response to a patient condition, verbalizing observed trends and their meaning to other team members), and anticipating (e.g., continually preparing for the next phase of the mission, setting and communicating intervention thresholds). Decision making referred to identifying options (e.g., recognizing alternative options for decisions, discussing clinical and other relevant considerations), balancing risks and selecting options (e.g. considering risks of different options, weighing up factors with respect to a patient’s condition), and re-evaluating (e.g., assessing a patient after key stages or regularly, reviewing a situation if the decision was to wait and see).
Nordby 2015 [45]	Norway	To present a case where the consequences of following a rule formulation “do not resuscitate” could have been fatal.	Qualitative: a case study	One case with two paramedics involved	To be able to interpret the validity of written guidelines, paramedics need to develop personal skills that transcend the ability simply to follow written instructions. Virtue ethical analyses, decision-making abilities, and communication skills are important as conceptual tools in making difficult clinical decisions.
Prytz 2018 [46]	Sweden	To investigate whether expert-novice differences in visual search behavior found in other domains also apply to accident scenes and the emergency response domain.	Quantitative: eye-tracking measurements, a task based on images presented on a computer screen	Students (n = 20), ambulance service staff (*n* = 9), rescue service staff (*n* = 8)	Expert emergency responders spent more time looking at task-relevant information (areas of the accident images), and were more accurate in the assessment of the accident scene than novices. The longer time was due to a longer fixation duration rather than a large fixation count.
Reay 2018 [47]	Canada	To understand how paramedics conduct decision-making in the field, and to develop a grounded theory of paramedic decision-making in the prehospital setting.	Qualitative: interviews, observations	Paramedics (*n* = 13)	Paramedic decision making was described as adaptive (flexible and creative) to the specific context and consisted of three categories: constructing a malleable model of the situation (based on information supplied by the dispatcher and gathered from the scene before reaching the patient), continuously revising the model (based on patient and scene conditions and available resources), and situation-specific action (based on reasoning about treatment decisions, working with protocols, seeking medical advice initiating treatment). Two additional components, considering patient and personal safety, and considering extrication are associated with each category.
Salminen-Tuomaala 2015 [48]	Finland	To identify factors that influence care quality and patient safety in out-of-hospital emergency care as experienced by emergency care professionals.	Qualitative: interviews	Paramedics (*n* = 5), nurses (*n* = 3), hospital and ambulance attendants (n = 3), practical nurses (*n* = 2)	High-quality emergency care is patient-centered, equal, professional, individualized and holistic. It encompasses the following areas: prompt emergency care on the scene; prevention of patient deterioration; individualized holistic care; arranging safe follow-up care; supporting the coping of patients and families; securing the safety of patients and staff on the scene and in the ambulance.
Sedlár 2017 [49]	Slovakia	To identify situation assessment and decision making strategies of EMS physicians in routine and non-routine situations.	Qualitative: interviews	EMS physicians (*n* = 15)	Critical assessments and decisions were related to the patient’s medical condition, patient’s behavioral and psychological characteristics, physical environment, and other people’s behavioral and psychological characteristics.
Schultz 2012 [50]	United States	To identify a set of all-hazard disaster core competencies and performance objectives required by the specific target audience (emergency department nurses, emergency physicians, and out-of-hospital emergency medical services personnel).	Modified Delphi	Participants representing multiple academic and provider organizations (*n* = 22)	All-hazard disaster core competencies were organized into domains: recognition, notification, initiation and data collection (recognize a disaster is in progress, assess the situation, initiate the disaster plan, notify the appropriate persons); public health and safety (prevent and mitigate risks to self and others); clinical considerations (manage patients with presentations that commonly occur during specific types of disasters); special-needs populations (manage patients according to their specific psychosocial, medical, cultural, age, and logistic needs); critical thinking/situation awareness (synthesize information and formulate new plans).
Smith 2013 [51]	United States	To study the cognitive strategies used by expert paramedics to contribute to understanding how paramedics and the EMS system can adapt to new challenges.	Qualitative: audio-video recordings of 2 simulation scenarios	Paramedics (*n* = 10)	The more experienced paramedics made more patient assessments, explored a wider variety of presumptive diagnoses, and identified the medical problem earlier. They switched attention more between two patients and used their team partner more, and provided more advanced level care for both patients. Their patients arrived at the emergency department more prepared for specialized emergency care.
Suserud 2003 [52]	Sweden	To describe the scope and method of ambulance nurse assessment in pre-hospital emergency care.	Qualitative: interviews	Ambulance nurses (*n* = 6)	Before meeting patients, experienced nurses plan less for routine calls and consciously plan for unusually serious calls. But in planning and initial assessment, they emphasized the importance of an open approach. It means to be prepared, but not committed to any particular courses of action. In the initial assessment, they emphasized the importance of capturing situations at a glance and doing on-the-spot primary assessments to give a comprehensive picture of the scene.
Suserud 2003 [53]	Sweden	To describe the scope of ambulance nurse assessment in pre-hospital emergency care and show how it is made.	Qualitative: interviews	Ambulance nurses (*n* = 6)	According to nurses, balancing the demands of medicine and nursing care are the essential ingredients in pre-hospital emergency care. They emphasized the importance of a detailed assessment of the patient’s medical condition, needs, social and cultural background, and on that basis, to make appropriate medical and nursing decisions/actions.
Tavares 2013 [54]	Canada	To develop and critically appraise a global rating scale for the assessment of individual paramedic clinical competence at the entry-to-practice level.	Mixed: Development phase – a task analysis using multiple simulation-based performances and actual clinical cases, a focus group, a modified Delphi process. Appraisal phase – video-recorded simulations and assessments of performance using the developed rating scale	Task analysis: experts from a paramedic program (the number not stated) Focus group: practicing paramedic clinicians (*n* = 17) Modified Delphi process: experts in paramedicine (*n* = 9) Simulations: novice paramedic students (*n* = 25), entry-to-practice students (*n* = 36), active paramedics (*n* = 24)	The global rating scale has four relevant categories that are described through their required attributes: situation awareness (e.g., observing the whole environment, avoiding tunnel vision), history gathering (e.g. interpreting and evaluating findings while discriminating between relevant and irrelevant findings, a consideration for differential diagnosis), patient assessment (e.g., the ability to continue appropriate reassessment/detailed assessment as needed, a consideration for differential diagnosis), decision making (e.g., selecting an appropriate management plan and/or decision strategy, avoiding premature closure).
Torabi 2017 [55]	Iran	To describe the experiences of Iranian prehospital emergency personnel in the field of ethical decision-making.	Qualitative: interviews	EMS personnel (*n* = 15)	Ethical decision making is based on the assessment of the scene atmosphere (local cultural beliefs and values, characteristics of the mission, the patient’s or bystander’s expectations), assessment of patients’ condition and their family (patient and disease characteristics, family preferences and perspectives), and predicting outcomes of decision-making (determination of medical futility, detecting potential risks and threats, forecasting legal consequences).
Von Wyl 2009 [56]	Switzerland	To test whether technical skills and non-technical skills are assessable with satisfactory interrater reliability during a regular paramedic training.	Quantitative: statistical analysis of observer-based ratings, simulation	Paramedics (*n* = 30)	The checklist for the rating of non-technical skills contained team components. Leadership components were making decisions and talking about considerations, decisions, and results. Membership components were making verbal or non-verbal decisions and talking about consideration, decisions, and results.
Wihlborg 2014 [57]	Sweden	To elucidate the desired professional competence of the specialist ambulance nurse, according to the professionals.	Modified Delphi	Specialist ambulance nurses, ambulance services managers, medical managers, clinical teachers, university teachers, scientists, union representatives: 1st round (*n* = 38) 2nd round (*n* = 37) 3rd round (*n* = 37)	The desired competencies were related to following areas: generic abilities (e.g. being flexible and adaptive, performing work in a problem-solving and thoughtful way), professional judgment (e.g. assessing the patient’s situation in a holistic manner, considering ethical principles), and professional skills (e.g., performing medical treatment and nursing care, working according to guidelines and for enhanced patient safety).
Wireklint Sundström 2011 [58]	Sweden	To describe and analyze assessment in pre-hospital emergency care provided by professional carers in the ambulance services, both for critical and non-critical conditions.	Qualitative: observations, field notes, interviews	Paramedics (*n* = 6), registered nurses (*n* = 3), specialist ambulance nurses (*n* = 2)	It was found that openness to the situation and recognition of the patient’s lifeworld is an essential part of assessments. Assessments focused solely on a patient’s medical condition can be an obstacle to a full understanding of the individual. A caring assessment focused on the patient’s suffering and needs adds further dimensions to the objective data. Therefore, the inclusion of the patient perspective relieves suffering and enables more safe decisions.
Wireklint Sundström 2012 [59]	Sweden	To study how ambulance personnel prepare themselves for their everyday assignments and avoid making premature decisions.	Qualitative: observations, interviews	Paramedics (*n* = 6), registered nurses (*n* = 3), specialist ambulance nurses (*n* = 2)	EMS requires the personnel to expect the particular event, but to avoid being governed by predetermined statements, to switch focus with respect to actual situational priorities, to adapt to a changing situation, to make immediate decisions/actions, to assess the situation continuously, to anticipate what might happen, to act systematically and in a structured way.
Wyatt 2003 [60]	Australia	To examine the utilization of tacit knowledge by experienced paramedics made explicit through their application of professional judgment.	Qualitative: observations, interviews	Mobile intensive care paramedics (*n* = 3)	Experienced paramedics are open to a variety of causes for the patient’s clinical problem, prepared not to have to arrive at a specific diagnosis, able to absorb a variety of information sources concurrently, direct attention to the problem at hand, make quick and sound judgments, choose whether to act or not, recognize multiple means and ends to clinical problems, discuss issues to arrive at a suitable course of action, and tend to rely less on established rules and guidelines.

### Situation awareness

The importance of developing a good awareness of a situation represents a common theme of many studies. Prehospital emergency situations, which can change very fast and at any moment, frequently require the development of situation awareness, both rapidly and on-the-spot [53], while synthesizing information about one’s surroundings into a big picture/model of the situation [34, 43, 44, 47, 50, 54]. Reay et al. [47] highlights constructing a malleable/flexible model, determined to be continuously revised and adapted to a changing situation [59]. The synthesis combines information from focused assessments that are here separated, yet in EMS professionals’ minds, they tend to overlap. The main assessment is focused on child or adult patients [42], and their various medical conditions and problems [31, 33, 37, 39–41, 47–51, 53, 57–59]. However, patients should not be taken only through the prism of medical issues, as they are individual human beings with different characteristics coming into play. Therefore, it is appropriate to assess them holistically, including their psycho-socio-economic-cultural background [33, 37, 39–41, 48, 49, 53, 55, 57, 58]. In this process, EMS crew members should not neglect information from patients’ significant others [39, 48, 49, 55]. They can be valuable sources of information to manage the emergency situation to a satisfying conclusion. It also emerged that it was crucial for those in a high-risk EMS profession to assess the environment and the safety of patients, bystanders, and personnel on scene and in the ambulance [31, 33, 34, 38, 40, 41, 47, 48, 55, 57]. This means assessing clues and significant objects in the environment that can give a notion of what happens and to assess potential risks and threats.

#### Gathering information

During gathering information before arriving on the scene, it is emphasized to pay full attention to the initial information from the emergency dispatch center [35]. This can provide initial pertinent information to construct a malleable model of the call. As EMS crew members approach and enter the scene, looking for clues related to the patient’s situation in the environment can add information to the model [47]. It is also important to observe the scene [54] and note (state) any environmental hazards to the self and the patient [38]. On the scene, the necessity is to focus and concentrate [43] – what an expert can do quickly and directly to the problem at hand [60]. Usually, gathering information centers on history taking and physical examination of the patient [42], which should be conducted systematically [54, 57], promptly [41, 47, 48], effectively, thoroughly, appropriately to a given situation [54], and via perception and active/careful listening to information provided from the dialog with the patient [35, 36, 59]. Although the patient is a key information source, the information provided from bystanders’ behavior and communication [37] is irreplaceable, especially when the patient is unconscious or unable to talk. However, the reliability of such information may not always be accurate. Other relevant markers of gathering information are discriminating between relevant and irrelevant data, avoiding tunnel vision [54], summarizing gathered information after some intervals [35], and verifying information to increase reliability [34, 44], e.g., checking verbally provided information by examination [35]. Furthermore, expertise research stresses the positive function of switching attention more between patients when more than one patient is involved [51] and spending more time looking at task-relevant areas of an accident [46]. Since emergency situations can change any minute, monitoring [31] or conducting a frequent scan of the environment [44] is crucial as a marker of good information gathering.

#### Interpreting information

Based on the collected information, EMS crew members made interpretations that are part of their mental model of the call. Although making good interpretations of patient‘s medical conditions is considered to be one of the required attributes [42, 57], it is critical to evaluate findings while discriminating between relevant and irrelevant data, avoid premature closure [54], rely less on the initial diagnostic hypothesis [51], and verify it by generating and exploring a wider variety of hypothetical diagnosis – differential diagnosis [33, 36, 51, 54]. Studies highlight being open to a variety of causes for the patient’s clinical representation (to accept the general and not to have to arrive at a specific diagnosis) [60], the ability to absorb, process, and combine a variety of information and information sources simultaneously [35, 47, 60], and making quick, sound judgments [60]. When an element of incongruent information appears, the necessity is to reconcile information [34], or to look for consistency among various information sources and to question inconsistent variables [47]. More noticeable markers of good interpreting information are responding to changes in the patient’s state, verbalizing observed trends and their meaning to other team members, increasing frequency of monitoring in response to patient condition [44], and interacting with team members that enable to correct potential misunderstanding [35].

#### Anticipating future states

Good situation awareness calls for anticipating what might happen: being aware of the probable scene trajectory [31, 43, 54, 55, 59] and acting anticipatively [51, 60], which is necessary before and after arriving on the scene. In this regard, some kind of open-mindedness [43], or avoiding being governed by pre-made assumptions and prejudices, whilst at the same time expecting the known (usually the worst scenario) and the unexpected [39, 52, 58], seems substantial. Wireklint Sundström and Dahlberg [59] label this as being prepared for the unprepared, while Suserud et al. [52] calls it being prepared but not committed to any particular courses of action. Examples of positive behaviors related to anticipating future states are: keeping ahead of the situation with appropriate intervention, setting and communicating intervention thresholds, or continually preparing for the next phase of the mission [44].

### Decision making

Included research articles mention making decisions/actions based on situation awareness, both rapidly [52, 59, 60] and systematically [59]. These decisions are primarily about medical treatment and management [40, 41, 44, 47], using various medical equipment, devices, and drugs, and occurring mostly with high density during the on-scene treatment phase of an emergency call [41]. Moreover, there are decisions made about the management of labor and delivery [41], nursing care and support [39, 40, 48, 53, 57, 58], deterioration prevention [48], extrication and transport [41, 44, 47], and safety-related decisions focused on preventing and mitigating risks of all persons involved [33, 40, 47, 48, 50, 57].

#### Generating and considering options

The next phase after understanding the nature of the situation and the patient’s problem is called generating and considering options. The research found that experts recognize not only a suitable option or what needs to be done, but also when and where it needs to happen, using subtleties in interpersonal communication [32]. It may concern a decision about what treatment to initiate, and when and where to initiate it [47]. Furthermore, the studies revealed several important markers such as recognizing and considering multiple options to solve the problem [43, 60], considering risks and benefits [54] of different treatment and transport options, weighing up factors with respect to the patient’s condition, assessing time criticality associated with possible options [44], and recognizing contraindications/reasons to withhold therapy [41]. More observable are markers such as seeking medical advice initiating treatment [47], seeking input on various transport-related issues with all relevant parties [44], and objectively evaluating and discussing clinical and other relevant considerations with team members [44, 56] to arrive at a suitable course of action [51]. These communication interactions occur more frequently in non-routine situations since courses of actions are already known in routine situations [49, 60].

#### Selecting and implementing an option

Selecting and implementing an option can be found in several articles that discuss the importance of selecting a safe, effective, situation-specific, appropriately prioritized and timed management plan and/or decision strategy [33, 34, 47, 54, 56]. The research highlights that experts can decide and act at the right time and place [32] based on incomplete information as emergency situations often require [43, 60]. Overall, they provide care on an advanced level and prepare their patients better for specialized care in the emergency department [51]. Some identified positive markers of this decision making element are to state the decision to approach a patient (or not) depending on the assessment of the scene [38], request assistance from other authorities to ensure safety [48], establish a safe zone [31], to state other final decisions based on the discussed options, and to agree on these final decisions [38].

#### Reviewing outcome/decision

Another critical step in good decision making is to see how the situation has changed or if a decision was effective and led to desired results. In this sense, studies emphasize making more patient assessments, continuous assessments/reassessments of the patient, or reviewing the entire situation [33, 38, 47, 51, 59]. In particular, it is desirable when expectations are violated [37, 47, 54], after implementing a treatment plan [41], if the decision was to wait, after key stages of the transport or regularly [44]. Such reassessment informs EMS crew members about the patient’s evolving condition and other (new) emerging information. This should be accompanied by making a list of options [44] and flexible changes in the course of action when needed [41, 60]. When certain implemented options are not working, it is good to stop current intervention, state alternative approach/intervention, and begin to use a more appropriate course of action [38]. Some cases even require the ability to modify or create new courses of action [43] – a hallmark of expertise [32].

### Maintaining standards

Included studies suggest an interconnection of maintaining standards with cognitive skills. Standards represent official structured methods of responding to emergency situations. They can be national, regional, and local guidelines, protocols and policies [42, 57], such as diagnostic guidelines [36, 48], treatment guidelines [40], advance directives [45], specific safety policies [33, 44], but also ethical principles and law [33, 42, 50, 55, 57]. These standards can guide situation awareness and decision-making processes. For instance, diagnostic guidelines may assist in gathering and interpreting information, and treatment guidelines and ethical principles help in making proper final treatment decisions. The authors mention the necessity of adherence to standards [42, 57], or at least knowing them [40] to consider and follow them when appropriate [33, 42, 44, 50, 55, 57]. At an EMS crew’s competition, following diagnostic guidelines was found to be a key in the success and may eliminate the risk of selection bias [35, 36]. However, it must be said that the competition underlines primarily guideline-based performance. According to Wyatt [60], as opposed to novices, experienced paramedics tend to rely less on guidelines and do not follow them uncritically and word for word. Instead, they interpret guidelines and situations through their prism of experience. Besides, trusting certain guidelines may have fatal consequences, so EMS crew members must develop personal skills that transcendent the ability simply to follow guidelines [45].

## Discussion

In this literature review, key articles relevant to EMS crew members’ situation awareness and decision making were synthetized. The results demonstrate an increase in the study of these skills in EMS crew members in recent years when compared to the first-ever non-technical skills review [4], which covered seven papers and brought only brief descriptions without any details regarding aspects and behavioral markers of cognitive skills. The interest in the reviewed topic is shown in articles targeting partial questions regarding cognitive skills, or articles trying to encompass all relevant non-technical skills or competencies essential for EMS crew members.

### Markers of cognitive skills

Many articles discuss specific aspects relevant to the cognitive skills of EMS crew members, such as the need to holistically assess a wide range of cues and information, to make various health- and safety-related decisions, and to take EMS standards into account. However, only three articles developed rating tools containing behavioral markers of cognitive skills, such as specific acts and verbalizations, that could be considered as examples of good behavior. One focused solely upon air ambulance clinicians [44], one was developed for remote/rural settings prehospital care providers [38], and one mixed technical and non-technical categories into a rating scale of paramedic clinical competence with low observability of markers [54]. More articles described required competencies [33, 50, 57], attributes [42, 43], knowledge [40], and important decisions [41] or important aspects of cognitive work in the EMS setting [47, 59]. They primarily do not show behavioral markers, but essential aspects that should be reflected in performance. On the contrary, expertise research is rare according to the low number of included articles in the review, which resulted in the description of some characteristics of expert EMS professionals based on observations and performance ratings [35, 36, 46, 51]. In this way, such obtained characteristics are similar to non-technical, behavioral rating systems. A study also proved the observability of a paramedic’s expert performance during cardiopulmonary resuscitation, which was recognized based on the entire pattern of his behavior, the smoothness with which he works, and that it seems that he knows what he was doing [13]. This form of expertise represents accumulated experience and not following the rules usually taught to student paramedics. When it comes to the nature of the situation examined, only two articles are concerned with highly non-routine, mass-casualty situations [43, 50]. This alarmingly low incidence conflicts with high threats to patient safety, and the high importance of cognitive skills in such situations. The remainder of the articles points to the prevalence of examining situations with one or a few patients.

As suggested, even though each included article constitutes a piece in the jigsaw puzzle of aspects and markers of non-technical cognitive skills in EMS crews, there is still a need for research to fill in many blank spaces. Research questions should be asked to elicit positive behavior and expertise characteristics, as well as negative behavior, cognitive errors, and biases, and it should be particularly focused on situation awareness and decision making in routine and non-routine emergency situations. Such an approach can give a richer and deeper understanding of EMS crew members’ cognition in its entirety and complexity.

### Interconnection of cognitive and other skills

The relationships between cognitive skills and other skills were also revealed. At first, a link between situation awareness and decision making occurred implicitly or explicitly across many articles. One can say, situation awareness is fundamental since its quality directly affects the quality of decision making [15]. Second, maintaining standards commonly classified as the element of leadership [3] or task management [9] emerged as entering the situation awareness and decision-making processes, in which various forms of standards – such as guidelines or protocols – are followed or considered [36, 47]. Therefore, this element was included as an integral element of cognitive skills, just as in a crisis resource management rating scale, incorporating ABC protocol into the problem-solving skills category [61]. Third, the third level of situation awareness [12], projection, or anticipating future states seems to be very close to planning and preparing, as outlined in some papers [52, 59]. In this regard, Klein et al. [62] place the third level of situation awareness into a relationship with anticipatory thinking as a process of preparing for future events and distinguishes it from simply predicting what might happen. Fourth, there was an association of cognitive skills with some components of team leadership and membership [34, 56]. Although the cognitive skills of leaders, as represented in the vast majority of articles, are more complex and demanding, sometimes other team members must switch their responsibilities with the leader’s role as a result of team working. It means that all team members must develop the same cognitive skills, which is in line with team cognition markers that Salas et al. [63] united for all team members. Fifth, the findings suggested the necessity of good communication skills, used to gather information about the patient either through a dialog with the patient [35] or with other key informants [37, 47]. Communication interactions also help crew members to share and discuss information and considerations to arrive at a good understanding of the situation and decisions [44, 60]. At last, history taking and physical examination conducted for the determination of a diagnosis [54], as rather medical skills, appeared in articles without specific information about how to do it from a purely medical point of view. However, they imply the significance of detailed patient assessment for situation awareness and decision making; hence, they were used in the review. These relationships are unsurprising, since non-technical skills are known to be close to one another and to medical and technical skills as well [18, 19]. Unfortunately, precise descriptions of their mutual relations, influences and overlaps are rather scarce, representing another area that requires examination. An inspiration on the road to fill this research gap can be the visualization framework of macrocognitive functions, showing how they are interrelated and dynamically interacting [64].

### Limitations

To make the research method and selection process rigorous, there was an exhaustive study of existing literature on the topic of cognitive skills and systematic reviews and the inclusion of more full texts to assess their eligibility, all to ensure any important paper has not been left out. The objectivity was promoted by the use of one independent rater for reviewing 30% of records and full texts, and the quality indicator of included articles applied already in database searches was the inclusion of peer-reviewed articles. However, these points may be viewed as a potential weakness of this literature review. A more critical approach would possibly bring slightly different findings. Further, the limitation is the exclusion of non-English articles, book chapters, and gray literature, which could add more relevant information. Strict selection criteria that restricted the capture of too specific aspects and all human, situational, and organizational factors that influence performance limits the results, yet the review did not have such an aim. If necessary, these issues could be inferred from the results gained here or addressed in future studies and reviews. It is also likely that some findings from related medical specialties are directly or after some adaptations applicable in EMS crew members; they were not included to keep the review as clear cut as possible. Orientation toward various EMS crew members (e.g., paramedics, emergency medical technicians, ambulance physicians, or ambulance nurses) can seem to be a misleading step. Nevertheless, to put them together was intentional because non-technical cognitive skills as general skills should apply essentially to all who work in the role of prehospital emergency care providers. Naturally, they usually need different specific medical and technical skills sets, depending on the prehospital setting (rural or urban) and health problem; this study is not about reviewing these skills. However, differences in the required level of training, skill sets and mandated interventions between EMS organizations in different countries can limit the generalizability and transferability of findings. That is why being prudent in the utilization of findings is recommended for researchers and practitioners.

## Conclusion

To sum up, this review synthesized the body of literature on important aspects and behavioral markers of two non-technical cognitive skills of situation awareness and decision making in professionals who work in EMS crews throughout different countries. The results revealed an increased research interest in the issue of cognitive skills, several specifics relevant to the prehospital emergency setting, and the interconnection of cognitive skills with other skills categories. Most importantly, they indicate the need to examine cognitive skills further, since findings from included articles either had a limited number of observable markers or, overall, they were not comprehensive for EMS crew members. The research should be also conducted to develop a tool for assessing and training non-technical cognitive skills, which can consequently lead to the improvement of quality and safety in prehospital emergency care.

## Data Availability

All data generated and analyzed during this study are included in this review article. All articles included in this review article are available in relevant journals and proceedings.

## Abbreviations

EMS: Emergency Medical Services
PRISMA: Preferred Reporting Items for Systematic Reviews and Meta-Analysis
